# Validation of an FFF-MALS Method to Characterize the Production and Functionalization of Outer-Membrane Vesicles for Conjugate Vaccines

**DOI:** 10.1021/acs.analchem.2c01590

**Published:** 2022-08-25

**Authors:** Robert M. F. van der Put, Arnoud Spies, Bernard Metz, Daniel Some, Roger Scherrers, Roland Pieters, Maarten Danial

**Affiliations:** † Department of Chemical Biology & Drug Discovery, Utrecht Institute for Pharmaceutical Sciences, Utrecht University, P.O. Box 80082, NL-3508 TB Utrecht, The Netherlands; ‡ Intravacc, P.O. Box 450, 3720 AL Bilthoven, The Netherlands; § Wyatt Technology Corp., Santa Barbara, California 93117, United States; ∥ Wyatt Technology Europe, D-56307 Dernbach, Germany

## Abstract

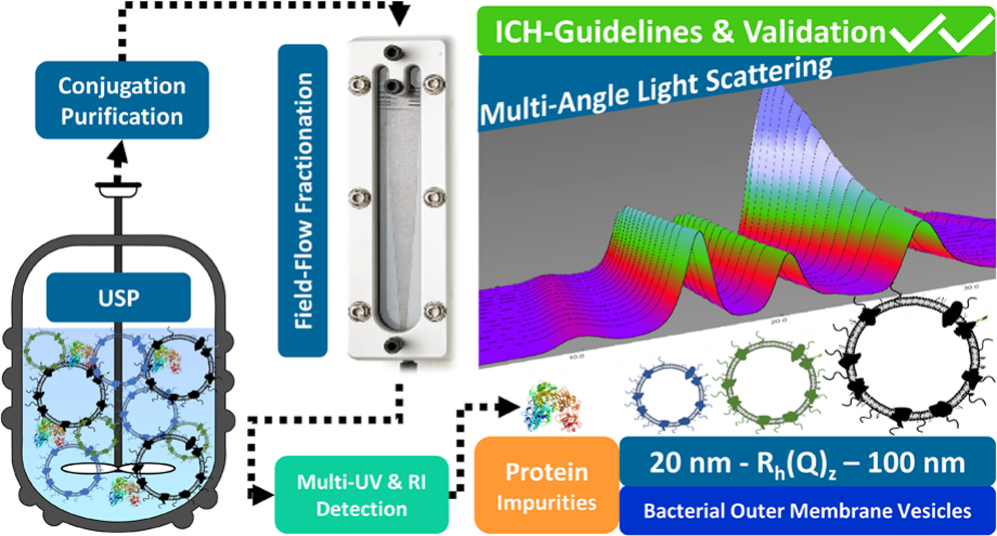

With the ongoing development of conjugate vaccines battling infectious diseases, there is a need for novel carriers. Although tetanus toxoid and CRM197 belong to the traditional carrier proteins, outer-membrane vesicles (OMVs) are an excellent alternative: in addition to their size, OMVs have self-adjuvanting properties due to the presence of genetically detoxified lipopolysaccharide (LPS) and are therefore ideal as a vaccine component or antigen carrier. An essential aspect of their development for vaccine products is characterization of OMVs with respect to size and purity. We report on the development of a field-flow fractionation multiangle light-scattering (FFF-MALS) method for such characterization. Here, we introduced NIST-traceable particle-size standards and BSA as a model protein to verify the precision of the size and purity analysis of the OMVs. We executed a validation program according to the principles provided in the ICH Guidelines Q2 (R1) to assess the quality attributes of the results obtained by FFF-MALS analysis. All validation characteristics showed excellent results with coefficients of variation between 0.4 and 7.32%. Estimation of limits of detection for hydrodynamic radius and particle concentration revealed that as little as 1 μg OMV still yielded accurate results. With the validated method, we further characterized a full downstream purification process of our proprietary OMV. This was followed by the evaluation of other purified OMVs from different bacterial origin. Finally, functionalizing OMVs with *N*-γ-(maleimidobutyryl)oxysuccinimide-ester (GMBS), generating ready-to-conjugate OMVs, did not affect the structural integrity of the OMVs and as such, they could be evaluated with the validated FFF-MALS method.

The development of conjugate vaccines against a variety of pathogens has been a cornerstone in disease prevention. This has been of particular importance for infants and children since the introduction of conjugate vaccines in the 1990s against pathogenic bacteria, such as meningococcus, *Haemophilus influenzae* type-b, and pneumococcus, resulting in a significant reduction in morbidity in Europe.^1^ These conjugate vaccines utilize carrier proteins^2^ like tetanus toxoid,^3^ diphtheria toxoid,^4^ the genetically modified cross-reacting material of diphtheria toxin (CRM_197_),^5^ meningococcal outer-membrane protein complex,^6^ or *H. influenzae* protein D.^7^ With the ongoing development of new conjugate vaccines targeting a large array of infectious diseases, there is a growing need to find alternatives for these traditional carriers. Outer-membrane vesicles (OMVs), spherical lipid bilayer membranes extracted from bacteria, would be an excellent option as these carriers are larger and thus bear more potential covalent linking sites relative to the smaller protein carriers. In addition, the size of OMVs offers a favorable trade-off between accumulation in draining lymph nodes on one hand and a high level of opsonization activity leading to an enhanced Th1 response on the other.^8,9^ OMVs are stable and permit ample opportunity for covalent conjugation of pathogen-specific antigens to membrane-associated proteins using water-compatible chemistries. Furthermore, OMVs are self-adjuvanting due to the presence of LPS within the membrane.^10^ While LPS is known to cause severe inflammation and can result in septic shock,^11^ efforts in the past 20 years have established detergent-enabled purification processes or genetic detoxification methods.^12^ These methods reduce the adverse effects of LPS, while preserving an adequate response to pathogen-associated molecular patterns, such as those recognized by the toll-like receptors TLR2 and TLR4.^13,14^ In particular, OMVs equipped with genetically detoxified LPS enable effective vaccine formulations without aluminum-based adjuvants,^15,16^ as opposed to many of the traditional conjugate vaccines. Other major advantages of OMVs are (1) they are highly amenable to genetic alteration or enhancement, so that unwanted proteins can be deleted or edited, creating a more favorable immunological profile toward the antigen of choice and (2) heterologous proteins originating from other high-risk or hard-to-produce pathogens (*e.g*. bacteria, viruses, or parasites) can be expressed. Both properties aid in the versatility and broad application of such OMVs as potential carriers for conjugate vaccines.

This combination of characteristics indicates the versatility of OMVs for use either in stand-alone drug products or as novel carriers for the next generation of conjugate vaccines consisting of pathogen-derived antigens such as extracted polysaccharides, synthetic oligosaccharides, peptides, or proteins.

En route to developing OMV-based conjugate vaccines, it is imperative to evaluate and characterize both purified and GMBS functionalized OMVs. Such an evaluation should confirm, on the one hand, the purity of OMV at the end of the production process, and on the other that functionalization using GMBS—enabling conjugation to any thiol-bearing immunogenic moiety—does not affect the integrity and size distribution of the OMV. Furthermore, such methods could be used to evaluate the progression of purification or downstream processing (DSP) in terms of purity and yield.

Purified OMVs are generally characterized by dynamic light scattering (DLS) or nanoparticle tracking analysis (NTA). DLS is predominantly used to assess the hydrodynamic radius of particles, but in most cases does not determine particle concentration and suffers from low resolution with respect to size distributions in mixed populations. NTA has the advantage that it can count particles and quantify particle-size distributions more accurately; however, the need for extremely large dilutions and the interference of impurities make it potentially unreliable.^17^ Additionally, the cutoff radius for NTA lies in the order of 30 nm, which makes it unsuitable for the OMVs we want to evaluate, which start at ∼25 nm. Finally, neither NTA nor DLS apply any separation to the sample, making both suboptimal for fully characterizing DSP samples with respect to purity in the presence of potential protein impurities.

Recent advances in particle characterization using field-flow fractionation (FFF) suggest that it is applicable both to characterizing the purification process of these OMVs and to determining the integrity of the intermediate and final conjugate vaccines. FFF is very productive for nanoparticle characterization when combined with multiangle light scattering (MALS) and additional detectors. Validation of an FFF-MALS method for characterizing liposomal drug formulations has been described by Parot et al.^18^ A fully optimized separation method makes FFF-MALS suitable not only for the characterization of purified and functionalized OMVs but also for DSP samples containing complex mixtures of impurities and OMVs. In addition, the method can quantify particle concentration more accurately than NTA because it separates the OMVs from the impurities, eliminating any potential cross-interference during detection.

In this study, we present optimization of the FFF separation and characterization of purified OMVs, a model impurity and particle standards by simulating their elution under different flow conditions. This initial method development was followed by a full validation of the FFF method according to current ICH guidelines (Q2 R1).^19^ Using the validated method, we evaluated the DSP of OMVs and several other purified OMV products. Finally, we evaluated GMBS-functionalized OMVs as a carrier for conjugate vaccines.

## Experimental Section

### FFF-MALS

The separation method used to characterize OMVs was a type of FFF known as asymmetric-flow field-flow fractionation, or AF4. The principle of AF4 is described in detail by Giddings.^20^ Given that AF4 is the most widespread and useful method of FFF, we commonly refer to it as FFF for simplicity. In brief, it is based on the application of two flow streams (crossflow and channel flow) in an open separation channel consisting of a solid plate parallel to a frit-supported membrane. The channel flow transports the sample through the channel, whereas the crossflow pushes the particles toward the membrane. Brownian motion counteracts the crossflow, causing the particles to diffuse away from the membrane in a size-dependent manner. As a result, smaller particles are on average higher above the membrane than larger particles. Since the channel flow is laminar and thus the flow velocity varies with the height above the membrane, the smaller particles encounter higher flow velocities due to their higher average height and are flushed out faster than larger particles, which remain closer to the membrane.

FFF is typically followed by multiple online detection modalities, including UV/vis, MALS, DLS, refractive index (RI), and/or fluorescence. These provide rich, high-resolution information on each size fraction generated during FFF.

### Field-Flow Fractionation Modeling

One means for modifying the separation properties of an FFF channel is the variation of the overall height of the channel using spacers of differing thickness. Another means is variation of the ratio of channel flow to crossflow, which may be done during the elution according to a preprogrammed method. The impact of varying the channel height, crossflow, and channel flow on the elution of particles of a given size may be predicted through numerical modeling and testing these effects *in silico* is very useful in developing an optimal FFF separation method. Such predictive modeling was performed using the SCOUT software (v R1705, Wyatt Technology—currently marketed under the product name VISION DESIGN), which applies first-principles FFF theory to calculate and display a predicted fractogram. The prediction includes possible band-broadening effects and the dilution of the sample in the FFF channel during separation. Iterating through a series of simulated conditions enables optimization of a method, and as a final optimization step, the results of a separation run may be fed back into SCOUT to adjust estimated physical parameters and come up with a final flow program.^21^ For modeling, we used assumed particle sizes between 50 and 100 nm. We adjusted the channel height and flow conditions to achieve elution of the OMV during the applied crossflow period to facilitate separation. FFF Methods A–D described below were developed in this way.

### FFF Separation Methods

For all of the described methods (A–D), a focus flow of 1.5 mL/min and an inject flow of 0.2 mL/min were applied to the short separation channel (Wyatt Technology). A Millipore 10 kDa molecular-weight cutoff (MWCO), regenerated cellulose membrane was installed in the channel along with spacers (both provided by Wyatt Technology). All crossflows were programmed using a linear decay.

#### Method A (OMV)

For Method A, a 350 μm wide-format spacer was installed in the channel. The carrier solvent was PBS (10 mM phosphate, 150 mM NaCl, pH 7.2) running at a detector flow rate of 1 mL/min. The elution method consisted of the following steps: Elution (0–3 min, 3 mL/min crossflow), Focus (3–4 min), Focus + inject (4–9 min), Focus (9–10 min), Elution (10–25 min, 3–0.1 mL/min crossflow), Elution (25–40 min, 0.0 mL/min crossflow), and Elution + Inject (40–45 min, 0.0 mL/min crossflow). The first two steps, though labeled here and in other methods as “Elution” and “Focus” in correspondence to the terms used in the FFF software, serve as channel flushes prior to sample injection.

#### Method B (OMV)

Method B was identical to Method A, apart from replacing the 350 μm spacer with a 250 μm wide-format spacer.

#### Method C (OMV-BSA)

For Method C, a 250 μm, wide-format spacer was used with 10 mM phosphate buffer, pH 7.2 as the eluent and a detector flow of 0.5 mL/min. The elution method consisted of the following steps: Elution (0–1 min, 3 mL/min crossflow), Focus (1–2 min), Focus + inject (2–4 min), Focus (4–6 min), Elution (6–11 min, 3 mL/min crossflow), Elution (11–16 min, 3–0.5 mL/min crossflow), Elution (16–34 min, 0.5–0.05 mL/min crossflow), Elution (34–40 min, 0.05 mL/min crossflow), and Elution + Inject (40–45 min, 0.0 mL/min crossflow).

#### Method D (Particle Standards)

For Method D, a 250 μm spacer wide format was used with 10 mM phosphate buffer, pH 7.2 as the eluent and a detector flow of 0.5 mL/min. The elution method consisted of the following steps: Elution (0–1 min, 1 mL/min crossflow), Focus (1–2 min), Focus + inject (2–4 min), Focus (4–6 min), Elution (6–11 min, 1 mL/min crossflow), Elution (11–16 min, 1–0.5 mL/min crossflow), Elution (16–34 min, 0.5–0.05 mL/min crossflow), Elution (34–40 min, 0.05 mL/min crossflow), and Elution + Inject (40–45 min, 0.0 mL/min crossflow).

### DLS and NTA

The description of these methods is available in the Supporting Information (Methods S3 and S4).

### ASTRA Data Processing

All light-scattering results described below were calculated using ASTRA software v. 7.3.2.19 (system 1) and/or 8.0.2.5 (system 2), both from Wyatt Technology.

#### Hydrodynamic Size

The online DLS module detects fluctuations in light scattered by particles in the MALS flow cell and provides autocorrelation functions (ACF) periodically, with a minimum ACF acquisition time of 2 s, to measure hydrodynamic radii across the fractogram. ASTRA determines diffusion coefficients from dynamic light scattering by employing the method of cumulants and then applies the Stokes–Einstein equation to determine the hydrodynamic radius *R*_h_(*Q*).^22^ The average radius across a peak or segment of the fractogram is calculated as the *z*-average of instantaneous radii ***R***_h_(*Q*) values, *R*_h_(*Q*)*_z_*.

#### Geometric Radius

The MALS detector quantifies the intensity of light, scattered by the sample in the flow cell, at 18 angles relative to the direction of propagation of the illuminating laser beam, at intervals of typically 0.5 or 1 s during the elution. The geometric radius *R*_geom_ is calculated from the angular dependence of the scattered light using a model that assumes a uniform sphere, and the average *R*_geom_ across a peak or segment of the fractogram is calculated as the *z*-average of the instantaneous values, *R*_geom,*z*_.^23^

#### Particle Concentration

Particle concentration *N* is calculated from MALS data.^24^ The refractive index of OMV used for determining *N* was initially set to 1.485 on knowledge that the OMV consists mostly of protein, and a spherical particle shape was assumed. The total number of particles in a peak or segment of the fractogram is calculated by integrating the product of the instantaneous particle concentration, the data collection interval, and the flow rate through the detector.

#### Molecular Weight

Calculation of molecular weight, used to validate BSA (Thermo Scientific, Pierce BSA, 23209) as an impurity, requires MALS and concentration data.^23^ Concentration was obtained from the refractive index detector, applying dn/dc of 0.185 mL/g for BSA and other proteins. The same dn/dc value was used in the MALS analysis.

### Chromatographic Parameters

All chromatographic parameters for the purity assessment of DSP fractions, only using FFF system 1, were calculated using Chromeleon software (v.7.2. SR 6 7553, Thermo Scientific), which was also used to control the instrument. Purity was calculated for different DSP fractions by comparing the UV^280^ peak area of the impurity compared to the total peak area of all eluting species. Considering the heterogeneity of the OMV population and inherent differences in the molar extinction coefficient, the purity assessment was taken as a qualitative parameter.

### OMV Production Process

The OMVs were produced as described previously^25,26^ and stored in a 10 mM TRIS buffer at pH 7.4 with 3% sucrose. For a concise overview of all DSP fractions, see Table 1.

**Table 1:** Overview of DSP Fractions for the Purification of OMVs

fraction	description
1	biomass after diafiltration
2	EDTA extracted biomass
3	after centrifugation of the extracted biomass
4	OMV after digestion
5	OMV after centrifugation
6	OMV after clarification/filtration
7	OMV after size exclusion chromatography
8	OMV after sterile filtration

### Validation Strategy

#### Accuracy

The accuracy of an analytical procedure expresses the closeness of agreement between the measured value and a value that is either the conventional “true” value or is otherwise an accepted reference value. For OMVs, there is no biological particle-size reference standard available (*e.g*. provided by National Institute of Standards and Technology (NIST) or another standards agency). However, polystyrene NIST-traceable nanospheres, available in a size range comparable to the OMV, were used for confirmation of the analytical FFF-MALS method. In addition, DLS and NTA measurements of the OMVs were used as a reference.

##### Particle Size

*R*_h_ of the OMV was first assessed six times (three times by two technicians) by both DLS and NTA. The *z*-average *R*_h_ from DLS, the number-average *R*_h_ from NTA, and CV (*n* = 6) from both assays were used for reference. This was followed by FFF-MALS analysis of the OMV six times (three times by two technicians), and *z*-average *R*_h_ and CV were calculated. Using the polystyrene size standards to show the accuracy for determining *R*_h_(*Q*)*_z_* in the range of the radii expected for the OMV (20–200 nm), these were assessed six times by FFF-MALS, and the average *R*_h_ and CV were calculated. A similar approach was applied to determine the value and CV of *R*_geom,z_.

##### Particle Concentration

For the assessment of particle concentration, the OMV was measured six times (three times by two technicians) by NTA to determine average and CV. FFF-MALS was performed six times (three times by two technicians) and average particle concentration and CV were calculated and compared to the NTA reference.

##### Impurity Profile

Evaluation of impurity profiles involved spiking different amounts of a model impurity, BSA, into an OMV sample, performing FFF separation, and quantifying the OMV size, OMV concentration, BSA molar mass, and eluted BSA mass. For the OMV, both DLS and NTA data served as a reference to verify the particle size (radius in nm) and concentration (particles/mL). Each measurement was performed over six repeats (three times by two technicians).

#### Precision

##### Repeatability

An OMV sample was analyzed at three different dilutions (undiluted, 2× and 4× diluted) in triplicate. Average *R*_h_ and particle concentration were determined, and from these the average and CV were calculated.

##### Intermediate Precision

The same experiment as for repeatability was performed by a second technician on a different day. New membranes were installed in the FFF channels and freshly prepared buffer applied. From these results, the average and CV between the two technicians were calculated.

##### Reproducibility

Reproducibility expresses the precision between the measurement results obtained at different laboratories. This interlaboratory variation was evaluated by comparing the particle standard analyses performed on FFF-MALS systems 1 and 2, both using Method D particle standards elution profile. From these results, the average and CV between the two laboratories were calculated.

#### Specificity

To ensure the identity of the analyte, three different batches of OMV were analyzed in triplicate. Additionally, particle standards in the size range of the OMV (20, 50, 100, and 200 nm) were analyzed 6-fold. Three blank runs were performed to demonstrate that no detector signal is observed in the elution range of the OMV and SSTs. Finally, BSA (67 kg/mol) was spiked into the OMV solution to show that free proteins do not coelute with OMV and that it was possible to separate free proteins from OMV (same run as purity assessment in the following section).

#### Purity Assessment

The OMV solution was spiked with BSA as a model protein to mimic the protein impurity and evaluated for recovery (Table S10). BSA did not interfere with OMV characterization since it did not elute in the range of protein impurities and was subsequently evaluated to the extent the spiked-in BSA could still be detected.

#### Linearity and Range

An effective dilution series was performed for OMV by decreasing the injected volume of the sample to a point that *R*_h_ or particle concentration could no longer be calculated. This assay was performed in triplicate. Five points were included to evaluate linearity. This determined the minimum quantity of OMV that could be injected while the analysis still yielded an accurate and precise result for hydrodynamic radius. Additionally, analysis of the particle concentration (particles/mL) over the dilution series should yield a calibration curve with the coefficient of determination *R*^2^ > 0.95.

#### Quantification and Detection Limit

Using the data from the linearity and range experiments, the limit of quantification and limit of detection were calculated.

#### GMBS Functionalization of OMVs

OMVs ∼1.4 mg/mL were buffer-exchanged to 10 mM HEPES pH 7.8 using manual hollow-fiber tangential-flow filtration (HF TFF, Repligen, C02-E100-05-S, 100 kDa MWCO). The OMV suspension was diluted to an effective protein concentration of 1.1 mg/mL, of which 2.25 mL was used for functionalization with GMBS (Mw 280.23 kDa) dissolved in DMSO. To facilitate GMBS reactions 1, 2, and 3, we dissolved 0.8, 1.2, and 1.8 mg GMBS, respectively, in 0.25 mL of DMSO and was subsequently added to the OMV. The reaction lasted for 30 min on a continuous roller bench at ambient temperature. The GMBS-modified OMVs were again buffer-exchanged to 10 mM HEPES pH 7.8 using manual HF TFF (Repligen, C02-E100-05-S, 100 kDa MWCO) before analysis.

## Results and Discussion

### FFF Method Development and Modeling

SCOUT software uses FFF theory to create *in silico*, “virtual” experiments that predict the fractogram resulting from a given set of conditions. The approximate particle sizes (*e.g*. obtained by offline DLS) are entered and the operator sets the channel-flow rate, crossflow profile (*i.e*., variation of crossflow with time, which may involve a crossflow gradient), spacer height, and channel type. To validate the FFF method, an initial method was developed using the modeling software to provide good separation conditions. This was followed by an optimization step.

An initial method assessment was performed using a standard default screening method (Method A). This was known to be a nonoptimal method, wherein the OMV might elute, in part, past the end of the crossflow gradient (Figure S1), where crossflow was set to zero, resulting in reduced resolution. Nevertheless, it was decided to evaluate the OMV elution behavior with this method, and we did in fact observe a pronounced delay in elution compared to the predicted elution according to the model developed in SCOUT: the OMV eluted fully past the end of the crossflow gradient (Figure S2). At that point, there is no longer size-dependent separation, and the eluting material consists of unseparated mixtures, which result in unreliable and unusable data.

The first iteration of the separation method involved changing the spacer height to 250 μm, per Method B. The benefit to changing spacer height was predicted by the *in silico* model, yielding earlier elution of the OMV, in the range where there would still be crossflow and subsequent separation (Figure S3). In practice, OMV elution was delayed once again beyond the crossflow gradient (Figure S4). It was hypothesized that the elution buffer (PBS), containing 150 mM NaCl, led to an interaction between the OMVs or of the OMV with the membrane. For a second iteration of the separation procedure, it was decided to continue using Method B but change the elution buffer to 10 mM phosphate, which now led to the OMV eluting well within the crossflow gradient.

Several further iterations on the crossflow gradient and the detector flow were made that facilitated elution of the individual particle-size standards, BSA and OMV (Figures S5 and S6). To facilitate the separation of BSA from OMV, an initial crossflow of 3 mL/min was found necessary for retaining the BSA so that it elutes after the void peak (which appears at ∼7.5 min). Ensuring that BSA elutes after the void peak was highly desirable when considering that the envisioned application is evaluation of DSP samples containing primarily free-protein impurities.

**Figure 1:**
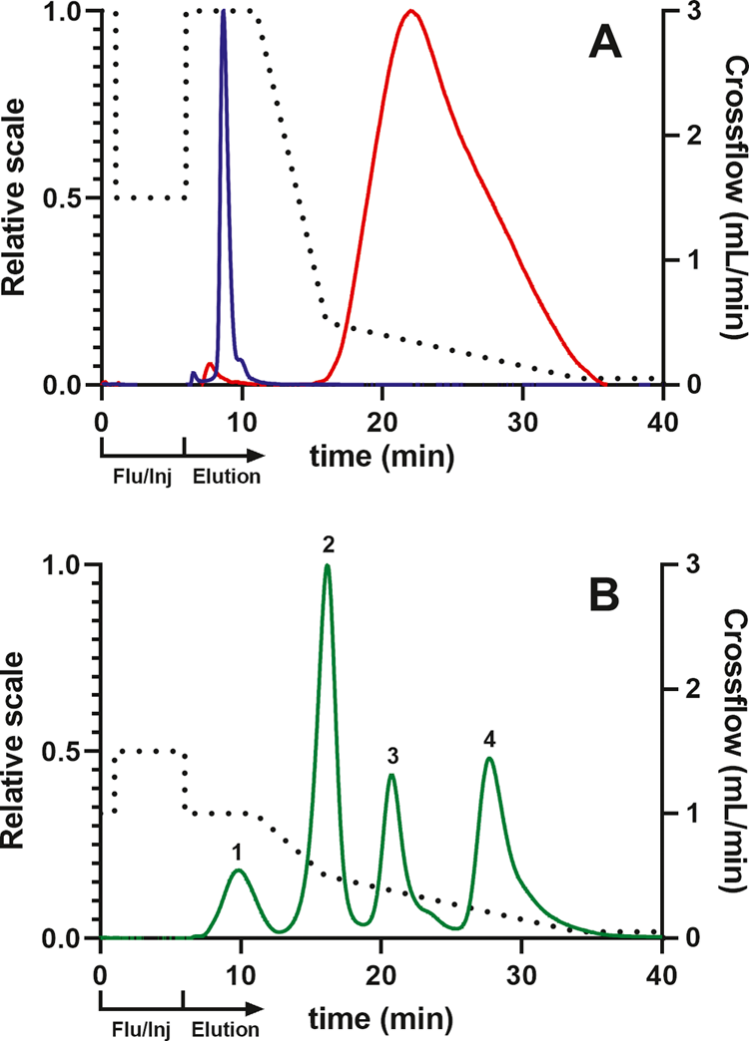
Elution profiles for the separation of BSA, OMV, and particle-size standards. The left-side y axes pertain to the relative signal from the 90° light-scattering detector. (A) Method C for the elution of BSA (blue) and OMV (red) and (B) Method D for the elution of particle-size standards (green) with radii of (1) 11.5 nm, (2) 25.5 nm, (3) 50.0 nm, and (4) 101.5 nm. The crossflow for both figures is plotted as the black dotted line, of which the first 6 min represent the steps to flush the channel and inject the sample (Flu/Inj).

This method was observed to work well for separating OMV and BSA. However, the particle-size standards could not be separated efficiently when an initial crossflow of 3 mL/min was applied. A method that separates these particle-size standards is required not only during the validation study but also as a system suitability test for a GMP-grade quality-control regime. After careful consideration, we ended up with two methods that differed only in the early elution program for retaining BSA but were the same for the region in which the OMV and particle-size standards eluted. Hence, we adopted two final elution profiles: one to separate BSA and OMV (Method C OMV-BSA, Figure 1a) and the other for particle-size standards (Method D Particle standards, Figure 1b). With these, we were able to separate the different entities and apply them to validating this FFF-MALS method (Figure 1).

### Validation of the FFF-MALS Method

With the goal of utilizing the FFF method for the characterization of OMV, it was decided to perform a full validation of the assay. Validation ensures that the assay and the results thereof may be relied upon in the analysis of drug substance following the production purification process, other purified OMV drug substances, GMBS-functionalized OMV, and possibly future conjugate vaccines employing OMV as a carrier. Furthermore, a validated FFF-MALS assay could be employed as a quality-control release assay and for stability studies of the OMV drug substance (concentrated bulk product) and drug product (final formulated vaccine, not part of this investigation).

ICH guidelines Q2 (R1)^19^ were evaluated and used in developing the validation plan. These guidelines state that particle-size determination for drug substances has not been addressed in the initial text on the validation of analytical procedures. In the absence of specific guidelines for particle-size determination, it was decided to validate according to the “testing for impurities” regime that includes all relevant characteristics: accuracy, precision, specificity, limit of quantitation, limit of detection, linearity, and range. Since the ICH guidelines do not state any limits and no known references were available in the field toward validation of a similar method, we did not set any predefined limits prior to validation of the analytical procedure.

With respect to particle-size standards, it was somewhat surprising that no biological standard, preferably NIST-traceable, was available. Therefore, nanosphere size standards, a polystyrene equivalent to OMV, were used instead to validate separation and analysis of particles in the size range of our OMVs. Additionally, we confirmed that the FFF method elutes particles of a known size at the elution time designated according to *in silico* modeling. With this, we were able to validate the method despite the absence of such a biological standard. With NIST-traceable particle-size standards and BSA defined as a model impurity, both Method C (OMV-BSA) and Method D (particle standards) were applied to the validation strategy outlined above.

#### Accuracy

For the validation of accuracy in particle size, the hydrodynamic radius of OMV was assessed by FFF-MALS, DLS, and NTA (Table S1). It was noted that there were inherent differences in the outcome of the individual methods, for example, batch DLS provides a harmonic *z*-average, NTA provides a number average, and FFF-MALS provides a *z*-average (though the latter can also provide number and mass averages).

Particle-size standards, in the range of the radii expected for the OMV, were also evaluated by FFF-MALS, DLS, and NTA (Tables S2–S4). For both DLS and NTA, individual particle standards were analyzed, but measurements of the mixture of sizes resulted in nondistinguishable individual peaks and very high polydispersity index and were hence not usable. Here, the advantage of the FFF really stood out as it produced useful data for each individual size after separation of the mix of particle standards. The 51 nm standard showed a slight offset in the final MALS result of around 60 nm confirmed by DLS and NTA. The NTA instrument was not able to determine the size of the 23 nm size standard, as expected, since the low cutoff for this analysis is around 30 nm.

Accuracy of particle concentration measurements for OMV was assessed with FFF-MALS and NTA (see Table S5). The measurement is not supported by the DLS instrument used in this investigation. There was a striking difference between the results from NTA (average 5.67 × 10^11^ particles/mL) and FFF-MALS (average 1.45 × 10^12^ particles/mL), where 2.6 times more particles were determined by FFF-MALS. This discrepancy could be ascribed to two factors: (1) the OMV distribution contains particles smaller than 30 nm, which are not detected by NTA but are included in the FFF-MALS analysis and (2) the RI value used for calculating the particle concentration in FFF-MALS (1.485) was estimated and is still under investigation.

Accuracy assessment for *R*_h_(*Q*)*_z_* and molecular weight of eluting BSA, envisioned as both a system suitability test and model impurity, showed excellent CV for both the molecular weight (CV 0.89%) and *R*_h_(*Q*)*_z_* (CV 2.50%) (see Table S6).

#### Precision

With regard to intermediate precision, the CV for both *R*_h_(*Q*)*_z_* (1.65%) and particle concentration (15.93%) was highest for the 4-fold diluted sample (see Tables S7 and S8). The intermediate precision (difference between technician 1 and technician 2) over three different dilutions was also calculated and resulted in a CV of 1.1% (see Tables S7 and S8).

#### Specificity

ICH guidelines prescribe that for assessing specificity, we are to ensure the identity of the analyte. In the absence of a biological reference standard, we chose to evaluate three batches of OMVs, analyzed in triplicate. Here, different elution profiles were observed for each OMV batch, but all eluted in the expected range (Figure S7 and Table S9). To further confirm that the method was specific for a particular range, particle standards in the size range of the OMV (20, 50, 100, and 200 nm) were assessed 6-fold (Figure S8). Blank runs performed in triplicate did not show any eluting particles (data not shown).

#### Purity Assessment

Purity was assessed by spiking BSA into OMV (Table S10). Baseline separation between OMV and BSA was observed for all spiked samples, excluding any potential matrix effects or interactions between the OMV and spiked BSA (Figure S9). Disregarding the differences in the molar extinction coefficient between BSA and OMV (UV^280^), this test shows that protein impurities were detected to a level of at least 1% w/w, when injecting 54 μg OMV or more. Furthermore, no specification was set for *R*^2^, but we could appreciate the excellent coefficient of 0.999 (Table S11 and Figure S10).

#### Linearity and Range

For the evaluation of linearity and range, a dilution series was performed on the OMV, in triplicate. The minimum quantity of OMV (μg) was evaluated by determining the point that *R*_h_ or particle concentration could no longer be quantified accurately. With respect to *R*_h_(*Q*)*_z_*, the minimum injected quantity that enabled determining *R*_h_ was as low as 1 μg (protein content of OMV). At 0.5 μg, the chromatograms became inconsistent, with subsequent CV going up to 15% (Table S12). While this test was not carried out, in principle, MALS is ∼100× more sensitive than online DLS and the limit of quantification for *R*_geom_ is expected to be about 0.01 μg (protein content of OMV).

Using the same dilution series, the particle concentration was determined, yielding a calibration curve with *R*^2^ > 0.980 (Figure S11). However, upon reviewing individual data points, it was observed that the CV % increased significantly and particle concentration became unreliable for injections containing 5 μg and less (Table S13).

#### Quantification and Detection Limit

#### Reproducibility

Interlaboratory variation was evaluated by comparing the results of particle standard analysis using the method. Here, it was observed that the two different laboratories, using two different FFF set-ups, produced comparable results in equivalence tests that fell within 10.5% (Tables S14 and S15).

#### Recovery

For both BSA and OMV, the recovery was evaluated and were 92.8 and 90.8%, respectively (Table S16).

#### Summary

We were able to successfully execute all experiments necessary to test the individual validation criteria. The size particle standards aided greatly, considering that a NIST-traceable biological standard representing OMV was not available. The successful repeated elution of a mixture of these standards gave a lot of confidence in the abilities of the method.

BSA was successfully introduced as a model protein to mimic impurities and was separated from OMV as demonstrated by FFF-MALS analysis. Before starting the validation, we had experience running and analyzing samples to a certain extent, not knowing how accurate the results could be. In conclusion, we were able to evaluate all validation criteria and were able to report the individual results. FFF-MALS analysis performed exceptionally well and showed very low CV % at all stages of the validation (see Table 2).

**Table 2:** Validation Results

validation parameter	determined limits (%CV)
accuracy—OMV *R*_h_(*Q*)*_z_*; Table S1	0.40
accuracy—particle standard SST geometric radius; Tables S2–S4	5.06^a^
accuracy—particle concentration; Table S5	7.32
accuracy—BSA SST (Mw); Table S6	2.50
intermediate precision—particle-size *R*_h_(*Q*)*_z_*; Table S7	1.10
intermediate precision—particle concentration; Table S8	1.10
repeatability—particle-size *R*_h_(*Q*)*_z_*; Table S7	1.65
repeatability—particle concentration; Table S8	4.03
purity; Tables S10–S11^b^	n.a.
LoD/LoQ—particle-size *R*_h_(*Q*)*_z_*; Table S12^b^	n.a.
LoD/LoQ—particle concentration; Table S13^b^	n.a.
reproducibility; Tables S14 and S15^c^	10.5^c^

a Highest CV found for 51 nm particle.

b Not based on CV.

c Highest value for the 51 nm particle.

### Evaluation of the OMV Downstream Purification Process

As stated in the ICH Q6B guidelines,^27^ knowledge of the physicochemical properties of the drug substance and drug product is desired when filing for approval. Product characterization and determining the size of the product as well as of the impurities (if present) are of essence to ensure product safety. In addition, ensuring product quality and consistency are of high importance within the downstream production process as well as at the drug substance and drug formulation stages. With the validated FFF-MALS method in hand, we now wanted to see if it was possible to evaluate the OMV DSP production process. To this end, fractions were collected at critical stages of the downstream production process and subjected to the validated FFF-MALS analysis. The spike experiments using BSA as a model impurity were particularly informative and it was of interest to see if the FFF-MALS method could be applied to complex matrices containing mixtures of impurities and OMVs.

**Figure 2:**
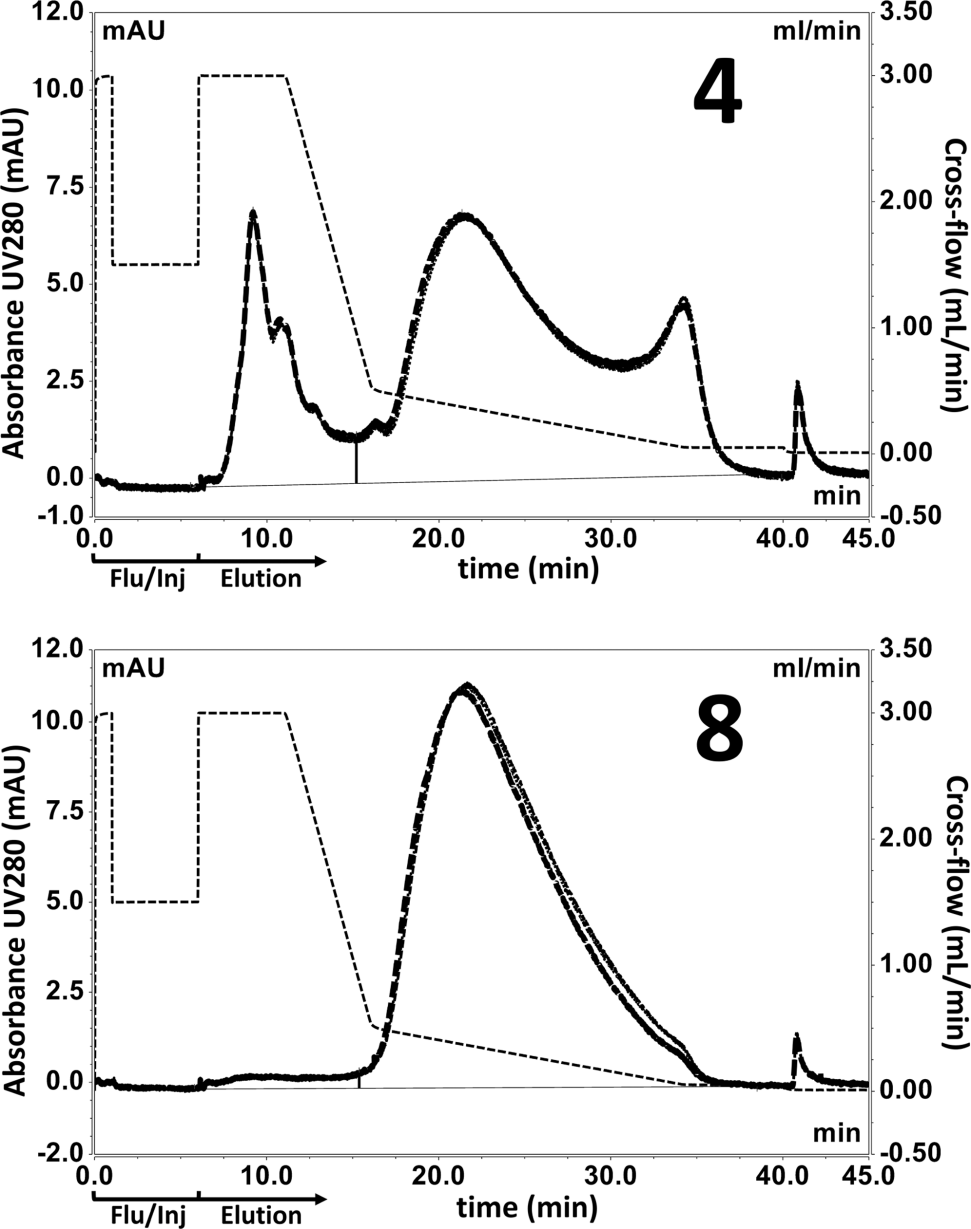
FFF-MALS results (*n* = 3): fractions 4 and 8 collected from DSP steps as described in Table 1 (for fractions 1–3 and 5–7), see Figure S12. Crossflow for both figures is shown as the black dotted line, of which the first 6 min represent the steps to flush the channel and inject the sample (Flu/Inj).

All fractions specified in Table 1 were analyzed in triplicate (Figures 2 and S12). For fraction 2, we did observe a slightly different elution pattern on one of the repeats, possibly attributed to the difficult matrix or the nonoptimal elution method for this fraction. However, it was well appreciated that other early fractions, from 1 to 6, also contain complex matrices, yet showed excellent comparability across repeats.

Purity was evaluated over the entire downstream process by separating and quantifying impurities relative to OMV. This evaluation is not without challenges. In cases where baseline separation between impurities and OMV did not occur in the UV fractogram, overlapping peaks were split at the bottom of the valley between them. Also, differences in molar extinction coefficients of the earlier eluting impurities can lead to either an over- or underestimation of the degree of purity. In early purification stages, some of the successive fractions did not show the expected increasing degree of purity, which we attribute to the difficult matrices and differing concentrations and volumes between those stages (Figures 2 and S12). Nevertheless, we were able to fully track all intermediate fractions and show that, after the final SEC purification step, the OMV product was 99.2% pure.

### Analysis of Different Purified OMVs

With a validated method in hand for characterizing OMV, we turned to investigating whether other types of purified OMVs could be evaluated by the same FFF-MALS method, Method C. This would be extremely helpful for the evaluation of new OMVs and conjugate vaccine carriers that are either extracted directly from the bacteria or are genetically constructed. The different purified OMVs consisted of *Neisseria meningitidis* type-B, *N. meningitidis* type-B containing two heterologous *Gonococcus* antigens, *Bordetella pertussis*, and *Escherichia coli*. These were produced using the same process as for the standard OMV product.

**Figure 3:**
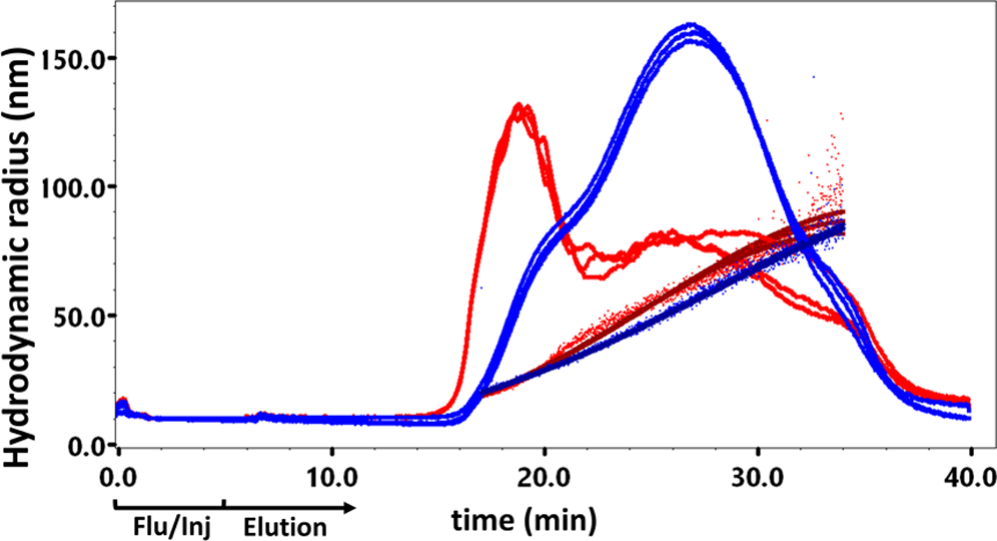
Different OMVs analyzed with FFF-MALS method C (OMV-BSA, *n* = 3). Blue: *B. pertussis*—*R*_h_(*Q*)*_z_* = 55.0 ± 0.4 nm; red: *E. coli*—*R*_h_(*Q*)*_z_* = 58.2 ± 0.7 nm.

All four of these OMVs were successfully eluted and analyzed using Method C OMV-BSA. Both *N. meningitidis* type-B OMVs showed an essentially similar fractogram as the OMV used in the validation study (data not shown). Delving into deeper detail, *e.g.*, in the *E. coli* OMV fractogram, we observe a distinct double peak, suggesting at least two size populations (Figure 3). For the *B. pertussis* OMVs, the fractogram also exhibited distinct differences, where we observed multiple populations (Figure 3). This demonstrates the advantage of FFF over low-resolution techniques such as DLS, where only a single broad peak with high polydispersity index would have been observed. Even though there were obvious differences in the elution profile between the different OMVs, the triplicates for each of the individual OMVs were in excellent agreement.

### GMBS Functionalization of OMVs

The use of GMBS for conjugating vaccine antigens to a carrier is a proven technology.^28,29^ The succinimide ester of GMBS targets primary amines, which are available as lysine residues on membrane proteins, phosphoethanolamine as part of LPS, or phosphatidylethanolamine as part of phospholipids, all of which are part of the OMV. Here, we investigated whether functionalization of OMVs using GMBS was possible without affecting the structure of the OMV. This functionalization would be a first step in preparation for any thiol-bearing antigen used in a succeeding conjugation step, suggesting a very broad range of applications.

**Figure 4:**
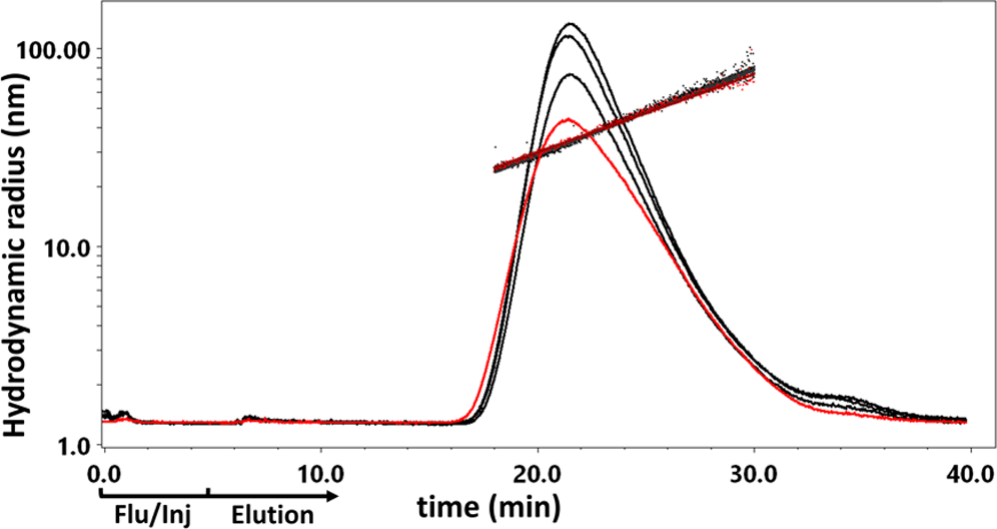
FFF-MALS analysis: OMV, OMV buffer-exchanged, and OMV-GMBS-modified; no differences in size distributions were found for OMV/GMBS ratios of 3:1, 2:1, or 1.3:1.

Other than some minor differences in the fractograms (differing peak areas due to different overall concentrations), functionalization with OMV/GMBS ratios of 3:1, 2:1, and 1.3:1 (w/w) did not affect the OMV size distribution (Figure 4). This information is highly beneficial for future conjugation chemistry approaches targeting a free thiol on an antigen.

## Conclusions

FFF-MALS methods were successfully developed to separate a model impurity, BSA, from OMVs and to separate a mixture of particle-size standards. Both these separation methods aided in the validation of FFF-MALS analysis of OMV. Where the ICH guidelines predominantly prescribed expected the result to fall within a CV of <30%, we observed surprisingly lower CVs for all evaluated parameters (see Table 2). This led to applying much lower CV requirements and, consequently, a higher quality level to the FFF-MALS analysis (Table 3). Recovery for both the model impurity BSA and OMV as target analyte was >90%, confirming the excellent quantitative performance of the analysis. Finally, it stood out that it was possible to evaluate the size and particle concentration of an OMV with as little as 1 μg of sample. This will be especially usable for evaluation of future down-scaled nonoptimized production processes during early process development.

**Table 3:** Validation Results

validation parameter	set limits (% CV)
accuracy—OMV *R*_h_(Q)*_z_*	<10
accuracy—particle standard SST geometric radius	<10
accuracy—particle concentration	<10
accuracy—BSA SST (Mw)	<10
repeatability—particle-size *R*_h_(Q)*_z_*	<10
repeatability—particle concentration	<20
purity	>1%^a^
intermediate—precision particle-size *R*_h_(Q)*_z_*	<10
intermediate—precision particle concentration	<20
reproducibility	<10
LOD—particle-size *R*_h_(Q)*_z_*	1 μg^a^
LOQ—particle-size *R*_h_(Q)*_z_*	1 μg^a^
LOD—particle concentration	1 μg^a^
LOQ—particle concentration	10 μg^a^

a Not based on CV.

With the validated method in hand, it was used to successfully evaluate the DSP process for the production and purification of OMVs. Even though the early fractions contain highly complex matrices, it was appreciated that all fractions could be evaluated for purity. Subsequently, different purified OMVs were successfully analyzed. Finally, the FFF-MALS method was used to evaluate the OMVs functionalized with GMBS in preparation for conjugation of any thiol-bearing vaccine antigen. Functionalization with different concentrations of GMBS yielded similar particle-size distributions. The OMVs held their integrity without decomposing or aggregating, which is essential for successful conjugate vaccine development. Further studies following the work presented in this paper will include the conjugation of synthetic oligosaccharides, synthetic peptides, and proteins. The application could potentially include antigens for a wide variety of infectious diseases (prophylactic), but therapeutic targets would also be of interest.

## Supporting Information Available

Results for all of the FFF-MALS validation criteria, SCOUT prediction models, and FFF-MALS fractograms, recovery measurements, method description for NTA, DLS, particle standards, FFF configurations, and safety statement (PDF)
