# Supplementary material for: Validation of an FFF-MALS Method to Characterize the Production and Functionalization of Outer-Membrane Vesicles for Conjugate Vaccines
Source: Anal Chem. 2022 Aug 25;94(35):12033–41. doi: 10.1021/acs.analchem.2c01590 (PMC9453738; doi:10.1021/acs.analchem.2c01590)
Supplement: Supplementary file 1 — ac2c01590_si_001.pdf [file ac2c01590_si_001.pdf]

Supporting Information

Validation of an FFF-MALS method to characterize the production and functionalization of outer membrane vesicles for conjugate vaccines.

Robert M. F. van der Put,[a,b*] Arnoud Spies,[b] Bernard Metz,[b] Daniel Some,[c] Roger Scherrers,[d] Roland Pieters,[a] Maarten Danial,[b]

[a] Department of Chemical Biology & Drug Discovery, Utrecht Institute for Pharmaceutical Sciences, Utrecht University, P.O. Box 80082, NL-3508 TB Utrecht, The Netherlands

[b] Intravacc, P.O. Box 450, 3720 AL Bilthoven, The Netherlands.

[c] Wyatt Technology Corp., Santa Barbara, CA 93117, USA

[d] Wyatt Technology Europe, D-56307, Dernbach, Germany

[*] Corresponding author: r.m.f.vanderput@uu.nl

TABLE OF CONTENTS

Table S1	Validation results for accuracy OMV	S3
Table S2	Validation results for accuracy particle size standards FFF-MALS	S3
Table S3	Validation results for accuracy particle size standards NTA	S3
Table S4	Validation results for accuracy particle size standards DLS	S4
Table S5	Validation results for accuracy particle count OMV FFF-MALS NTA	S4
Table S6	Validation results for accuracy	S4
Table S7	Validation results for intermediate precision OMV Rh FFF-MALS	S5
Table S8	Validation results for intermediate precision OMV particle count FFF-MALS	S5
Table S9	Validation results for specificity OMV FFF-MALS	S5
Table S10	Validation set-up for OMV spiked with BSA FFF-MALS	S6
Table S11	Validation results for OMV spiked with BSA FFF-MALS	S6
Table S12	LOD LOQ Rh(Q)z OMV FFF-MALS	S6
Table S13	LOD LOQ particle count OMV FFF-MALS	S7
Table S14	Particle size standards evaluated at Wyatt technologies Dernbach	S7
Table S15	Equivalence TOST-test	S7
Table S16	Recovery	S7
Figure S1	Scout prediction method A	S8
Figure S2	Separation OMV using method A	S8
Figure S3	Scout prediction method B	S8
Figure S4	Separation OMV using method B	S9
Figure S5	Scout prediction method C OMV-BSA	S9
Figure S6	Separation OMV using method C OMV-BSA	S9
Figure S7	Validation results for specificity OMV FFF-MALS	S10
Figure S8	Elution of particle size standards elution using method D particle standards	S10
Figure S9	Validation results for OMV spiked with BSA FFF-MALS	S10
Figure S10	Validation results for OMV spiked with BSA FFF-MALS	S11
Figure S11	LOD LOQ particle count OMV FFF-MALS	S11
Figure S12	Fraction 2, 5, 6 and 7 from the downstream purification process	S12
Method S1	FFF-MALS configurations	S13
Method S2	BSA and particle standards	S13
Method S3	Nanoparticle tracking analysis	S13
Method S4	Batch dynamic light scattering	S14
General	Safety statement	S14

I. Supporting information Tables

Table S1. Accuracy: OMV average radius, measured by DLS, NTA and FFF-MALS

Repetition	R_h from DLS (harmonic z-average radius, nm)	Mean size from NTA (number-average radius, nm)	$R_h(Q)_z$ from FFF-MALS (z-average hydrodynamic radius, nm)
**1 (Technician 1)**	49.0	65.7	54.0
**2 (Technician 1)**	49.0	62.4	53.8
**3 (Technician 1)**	48.4	60.5	54.1
**4 (Technician 2)**	48.6	60.2	53.6
**5 (Technician 2)**	48.4	60.6	54.0
**6 (Technician 2)**	48.7	61.4	53.6
**Average**	48.7	61.8	53.9
**Std. deviation**	0.3	2.1	0.2
**CV (%)**	0.56	3.32	0.40

Table S2. Accuracy: Z-average geometric radius $R_{geom,z}$ of a mixture of particle size standards, separated and measured by FFF-MALS

Repetition	Peak 1 (nominal size: 11.0 nm) $R_{geom,z}$ (nm)	Peak 2 (nominal size: 25.5 nm) $R_{geom,z}$ (nm)	Peak 3 (nominal size: 51.0 nm) $R_{geom,z}$ (nm)	Peak 4 (nominal size: 101.5 nm) $R_{geom,z}$ (nm)
**1 (Technician 1)**	11.0	29.0	51.1	101.1
**2 (Technician 1)**	11.0	30.2	51.5	101.6
**3 (Technician 1)**	11.0	27.8	50.5	101.0
**4 (Technician 2)**	10.2	32.1	52.7	102.0
**5 (Technician 2)**	11.0	28.7	50.9	101.0
**6 (Technician 2)**	10.8	29.2	51.0	100.8
**Average**	10.8	29.5	51.3	101.3
**Std. deviation**	0.3	1.5	0.8	0.5
**CV (%)**	2.96	5.06	1.49	0.45

Table S3. Accuracy: Mean radius of mixtures of individual particle size standards, measured by NTA

Repetition	Mean size (nominal: 11.5 nm)	Mean size (nominal: 25.5 nm)	Mean size (nominal: 51.0 nm)	Mean size (nominal: 101.5 nm)
**1 (Technician 1)**	n.d.	29.7	46.9	95.8
**2 (Technician 1)**	n.d.	32.2	46.9	96.8
**3 (Technician 1)**	n.d.	29.5	47.0	96.1
**4 (Technician 2)**	n.d.	29.5	47.2	96.0
**5 (Technician 2)**	n.d.	32.3	46.2	97.3
**6 (Technician 2)**	n.d.	36.9	46.8	93.6
**Average**	n.d.	31.67	46.83	95.92
**Std. deviation**	n.d.	2.9	0.3	1.3
**CV (%)**	n.d.	9.10	0.72	1.34

n.d. particle standards of 23 nm could not be measured by NTA.

Table S4. Accuracy: Harmonic z-average radius of individual particle size standards, measured by DLS

Repetition	Mean size (11.5 nm)	Mean size (25.5 nm)	Mean size (51.0 nm)	Mean size (101.5 nm)
**1 (Technician 1)**	11.9	31.6	56.8	109.8
**2 (Technician 1)**	11.8	31.3	56.9	111.4
**3 (Technician 1)**	11.7	30.9	55.4	110.4
**4 (Technician 2)**	11.6	30.9	55.9	109.8
**5 (Technician 2)**	11.8	31.0	56.0	111.4
**6 (Technician 2)**	11.8	30.8	56.0	111.4
**Average**	11.8	31.1	56.1	110.7
**Std. deviation**	0.1	0.3	0.6	0.8
**CV (%)**	0.79	0.99	1.02	0.73

Table S5. Accuracy: Overall particle concentration of OMV, measured by FFF-MALS and NTA

Repetition	Particles per mL (NTA)	Particles per mL (FFF-MALS) [1]
**1 (Technician 1)**	3.85×10^{11}	1.37×10^{11}
**2 (Technician 1)**	5.87×10^{11}	1.33×10^{12}
**3 (Technician 1)**	5.70×10^{11}	1.60×10^{12}
**4 (Technician 2)**	6.20×10^{11}	1.47×10^{12}
**5 (Technician 2)**	6.18×10^{11}	1.55×10^{12}
**6 (Technician 2)**	6.21×10^{11}	1.38×10^{12}
**Average**	5.7×10^{11}	1.45×10^{12}
**Std. deviation**	9×10^{10}	1.1×10^{11}
**CV (%)**	16	7

[1] Particles n/mL= Particles (n)/30*1000

Table S6. Accuracy: Weight-average molar mass M_w and z-average hydrodynamic radius $R_h(Q)_z$ of BSA, measured by FFF-MALS

Repetition	M_w (kDa)	$R_h(Q)_z$ (nm)
**1 (Technician 1)**	65.2	3.3
**2 (Technician 1)**	66.6	3.3
**3 (Technician 1)**	66.0	3.1
**4 (Technician 2)**	65.1	3.3
**5 (Technician 2)**	65.7	3.3
**6 (Technician 2)**	66.2	3.3
**Average**	65.8	3.3
**Std. deviation**	0.6	0.1
**CV %**	0. 9	2.5

Table S7. Intermediate precision: z-average hydrodynamic radius $R_h(Q)_z$ of OMV, measured over 3 dilutions by FFF-MALS

Repetition	Undiluted $R_h(Q)_z$ (nm)	2-fold diluted $R_h(Q)_z$ (nm)	4-fold diluted $R_h(Q)_z$ (nm)
**1 (Technician 1)**	54.0	53.4	54.7
**2 (Technician 1)**	53.8	53.5	53.4
**3 (Technician 1)**	54.1	53.6	55.0
**4 (Technician 2)**	53.6	53.5	52.8
**5 (Technician 2)**	54.0	53.8	54.3
**6 (Technician 2)**	53.6	53.8	53.2
**Average**	53.9	53.6	53.9
**Std. deviation**	0.2	0.2	0.9
**CV %**	0.4	0.3	1.7

Table S8. Intermediate precision: Particle concentration of OMV, measured over 3 dilutions by FFF-MALS

Repetition	Undiluted Particles/mL	2-fold diluted Particles/mL	4-fold diluted Particles/mL
**1 (Technician 1)**	1.37×10^{12}	5.77×10^{11}	1.76×10^{11}
**2 (Technician 1)**	1.33×10^{12}	6.93×10^{11}	2.40×10^{11}
**3 (Technician 1)**	1.47×10^{12}	5.87×10^{11}	1.91×10^{11}
**4 (Technician 2)**	1.60×10^{12}	6.03×10^{11}	2.57×10^{11}
**5 (Technician 2)**	1.55×10^{12}	5.33×10^{11}	2.52×10^{11}
**6 (Technician 2)**	1.38×10^{12}	5.63×10^{11}	1.98×10^{11}
**Average**	1.45×10^{12}	5.9×10^{11}	2.2×10^{11}
**Std. deviation**	1.1×10^{11}	5×10^{10}	3×10^{10}
**CV %**	7	9	16

Table S9. Specificity: z-average hydrodynamic radius $R_h(Q)_z$ for 3 individual batches of OMV, measured by FFF-MALS

Repetition	Batch-1 $R_h(Q)_z$	Batch-2 $R_h(Q)_z$	Batch-3 $R_h(Q)_z$
**1 (Technician 1)**	57.0	53.6	50.5
**2 (Technician 1)**	57.1	54.0	50.2
**3 (Technician 1)**	56.8	53.6	50.8
**Average**	57.0	53.7	50.5
**Std. deviation**	0.2	0.2	0.3
%RSD	0.3	0.4	0.6

Table S10. Purity: Validation set-up for OMV spiked with BSA FFF-MALS

Spiked BSA (%)	OMV (µg) [1]	BSA (µg)	OMV (µL)	OMV (µg)	BSA (µg)	µL BSA	WFI (µL)
**1**	54.3	0.54	239	216	2.16	1.08	54.3
**2.5**	54.3	1.36	240	217	5.43	2.71	54.3
**5**	54.3	2.72	240	217	10.86	5.43	54.3
**10**	54.3	5.43	240	217	21.71	10.86	54.3
**15**	54.3	8.15	240	217	32.57	16.28	54.3
**20**	54.3	10.86	240	217	43.43	21.71	54.3
**25**	54.3	13.58	240	217	54.28	27.14	54.3
**50**	54.3	27.15	240	217	108.56	54.28	54.3

[1] Based on OMV at 0.9047 mg/mL total protein concentration (Peterson), injection volume 75 µL, yielding 54.3 µg OMV per injection

Table S11. Purity: Validation results for OMV spiked with BSA FFF-MALS

Spiked BSA + OMV (%)	1	2.5	5	10	15	20	25	50
**Peak Area (mAU*min) 1**	0.95	1.67	3.73	6.72	10.17	13.26	16.91	32.26
**Peak Area (mAU*min) 2**	0.96	1.68	3.80	7.01	10.78	13.91	17.40	33.55
**Peak Area (mAU*min) 3**	0.88	1.67	3.46	7.32	10.57	14.08	17.28	33.71
**Average**	0.93	1.67	3.66	7.0	10.51	13.8	17.2	33.2
**Std. deviation**	0.04	0.01	0.18	0.3	0.31	0.4	0.3	0.8
**%RSD**	4.4	0.4	5	4	3	3	1.5	2.4

Table S12. Data for determining LOD and LOQ of z-average hydrodynamic radius $R_h(Q)_z$ of OMV by FFF-MALS

Q OMV (µg)	0.5	1	2.5	5	10	15	20	25	50	75
**Size Rh(Q)z 1**	60.3	41.4	41.3	40.9	41.6	41.5	41.9	41.9	41.8	40.8
**Size Rh(Q)z 2**	54.1	41.7	40.6	41.4	41.6	41.6	41.8	41.5	41.9	40.6
**Size Rh(Q)z 3**	44.2	40.8	41.4	40.9	41.3	41.7	41.7	41.6	41.6	40.3
**Average**	53	41.3	41.1	41.1	41.5	41.6	41.8	41.7	41.8	40.6
**Std. deviation**	8	0.5	0.4	0.3	0.2	0.1	0.1	0.2	0.2	0.3
**CV %**	15	1.1	1.1	0.7	0.4	0.2	0.2	0.5	0.4	0.6

Table S13. Data for determining LOD and LOQ of particle concentration of OMV by FFF-MALS

**Q OMV (µg)**	**0.5**	**1**	**2.5**	**5**	**10**	**15**	**20**	**25**	**50**	**75**
**Particle (n/mL) 1**	2.7×10^{8}	3.7×10^{9}	1.4×10^{10}	5.1×10^{10}	4.3×10^{10}	6.7×10^{10}	8.1×10^{10}	1.12×10^{11}	1.83×10^{11}	2.28×10^{11}
**Particle (n/mL) 2**	1×10^{7}	3.2×10^{9}	1.0×10^{10}	3.4×10^{10}	4.6×10^{10}	6.8×10^{10}	8.3×10^{10}	1.03×10^{11}	1.87×10^{11}	2.39×10^{11}
**Particle (n/mL) 3**	6.1×10^{8}	2.3×10^{9}	1.6×10^{10}	2.8×10^{10}	4.6×10^{10}	6.9×10^{10}	9.0×10^{10}	1.12×10^{11}	1.92×10^{11}	2.45×10^{11}
**Average**	3×10^{8}	3.1×10^{9}	1.3×10^{10}	3.8×10^{10}	4.5×10^{10}	6.80×10^{10}	8.5×10^{10}	1.09×10^{11}	1.87×10^{11}	2.37×10^{11}
**Std. Dev.**	23×10^{8}	7×10^{8}	3×10^{9}	1.2×10^{10}	1.8×10^{9}	6×10^{8}	5×10^{9}	5×10^{9}	5×10^{9}	9×10^{9}
**CV %**	100	22	24	31	4	0.9	5.7	4.7	2.4	3.6

Table S14. Reproducibility: Z-average geometric radius $R_{geom,z}$ of mixed particle size standards evaluated by FFF-MALS at Wyatt Technology

	**Peak 1 (nominal – 11.0 nm) $R_{geom,z}$ (nm)**	**Peak 2 (nominal – 25.5 nm) $R_{geom,z}$ (nm)**	**Peak 3 (nominal – 51.0 nm) $R_{geom,z}$ (nm)**	**Peak 4 (nominal – 101.5 nm) $R_{geom,z}$ (nm)**
**1**	12.1	27.9	54.7	100.0
**2**	12.6	28.3	56.0	99.5
**3**	10.5	27.5	55.7	99.3
**4**	11.5	28.8	57.0	99.2
**5**	10.3	28.7	56.0	98.6
**6**	10.3	30.3	55.7	98.7
**Average**	11.2	28.6	55.9	99.2
**Standard deviation**	1.0	1.0	0.7	0.5
**CV%**	8.89	3.40	1.32	0.52

Table S15. Reproducibility: Equivalence TOST-test

	**Equivalent (TOST-test)***
**Peak 1 (11.0 nm)**	Yes (p=0.047)
**Peak 2 (25.5 nm)**	Yes (p=0.008)
**Peak 3 (51.0 nm)**	Yes (p=0.018)
**Peak 4 (101.5 nm)**	Yes (p<0.0001)

*Threshold: 10.5%

Table S16. Recovery calculations for BSA and OMV

	**BSA A_S (mAU/min)**	**BSA A_D (mAU/min)**	**OMV A_S (mAU/min)**	**OMV A_D (mAU/min)**
**Measurement 1**	48.8	52.2	73.7	48.8
**Measurement 2**	46.1	52.4	71.4	46.1
**Measurement 3**	49.4	51.1	71.5	49.4
**Average**	48.1	51.9	72.2	48.1
**Recovery (%)**	**92.6**		**90.8**	

A_S is the peak area of the eluted sample, A_D is the peak area of sample directly injected into the detector. Analyte recovery (%): A_S/A_D*100.

II. Supporting information Figures

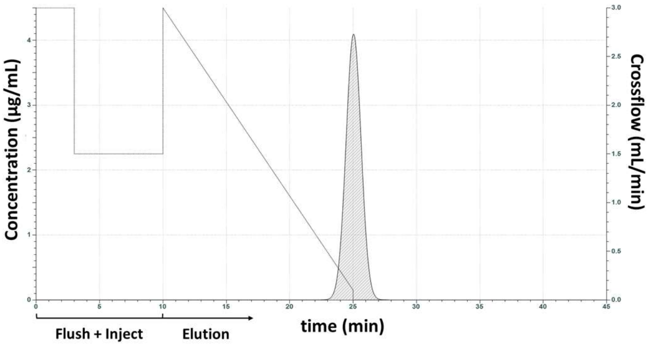

Fig. S1. Fractogram predicted by SCOUT FFF method development software, for Method A, assuming a wide format, 350 µm spacer and 1 mL/min detector flow. The elution method consisted of the following steps: Elution (0 - 3 min, 3 mL/min cross flow), Focus (3 - 4 min), Focus + inject (4 – 9 min), Focus (9 - 10 min), Elution (10 – 25 min, 3 – 0.1 mL/min crossflow gradient), Elution (25 – 40 min, 0.0 mL/min cross flow), Elution + Inject (40 – 45 min, 0.0 mL/min cross flow).

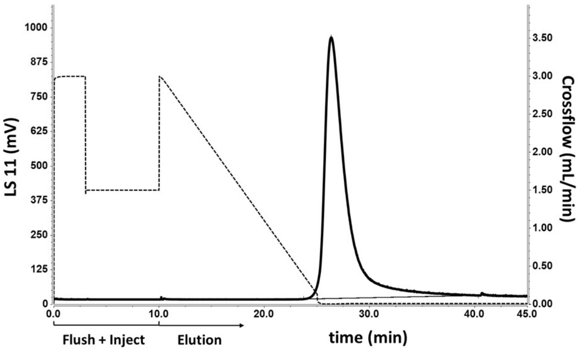

Fig. S2. UV fractogram from Chromeleon for OMV eluting with **Method A**: x-axis: time (min), y-axis left: LS 11 signal (mV), Y-axis right: Cross flow (mL/min).

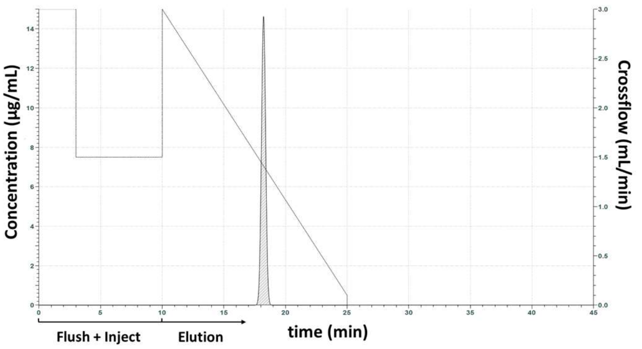

Fig. S3. Fractogram predicted by SCOUT FFF method development software, for Method B, assuming a wide format, 250 µm spacer and 1 mL/min detector flow. The elution method consisted of the following steps: Elution (0 - 3 min, 3 mL/min cross flow), Focus (3 - 4 min), Focus + inject (4 – 9 min), Focus (9 - 10 min), Elution (10 – 25 min, 3 – 0.1 mL/min crossflow gradient), Elution (25 – 40 min, 0.0 mL/min cross flow), Elution + Inject (40 – 45 min, 0.0 mL/min cross flow).

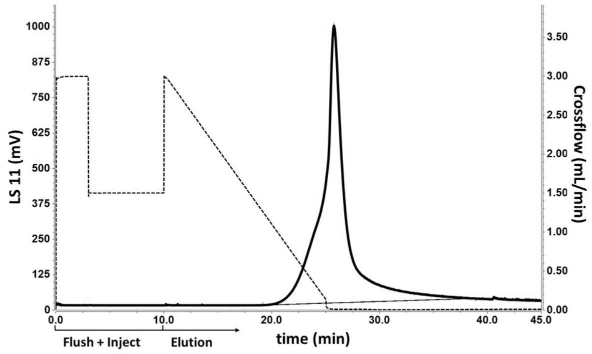

Fig. S4. UV fractogram from Chromeleon for OMV eluting with **Method B**: x-axis: time (min), y-axis left: LS 11 signal (mV), Y-axis right: Crossflow (mL/min).

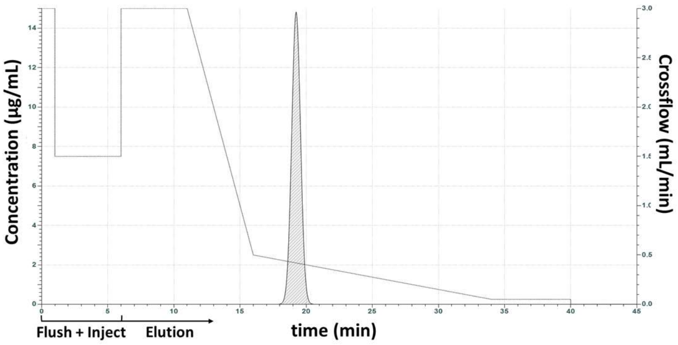

Fig. S5. Fractogram predicted by SCOUT FFF method development software, for Method C, assuming a wide format, 250 µm spacer and 1 mL/min detector flow. The elution method consisted of the following steps: Elution (0 - 1 min, 3 mL/min cross flow), Focus (1 - 2 min), Focus + inject (2 – 4 min), Focus (4 - 6 min), Elution (6 – 11 min, 3 mL/min cross flow), Elution (11 – 16 min, 3 – 0.5 mL/min cross-flow gradient), Elution (16 – 34 min, 0.5 – 0.05 mL/min cross-flow gradient), Elution (34 – 40 min, 0.05 mL/min cross flow), Elution + Inject (40 – 45 min, 0.0 mL/min cross flow).

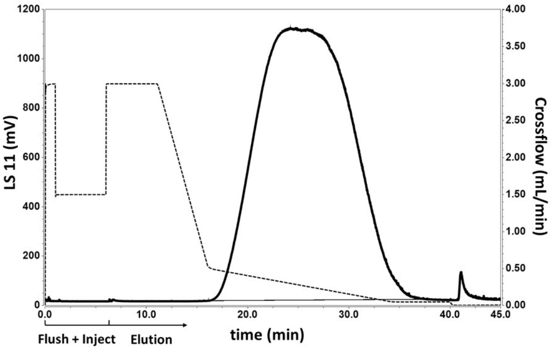

Fig. S6. UV fractogram from Chromeleon for OMV eluting with **Method C OMV-BSA**: x-axis: time (min), y-axis left: LS 11 signal (mV), Y-axis right: Crossflow (mL/min).

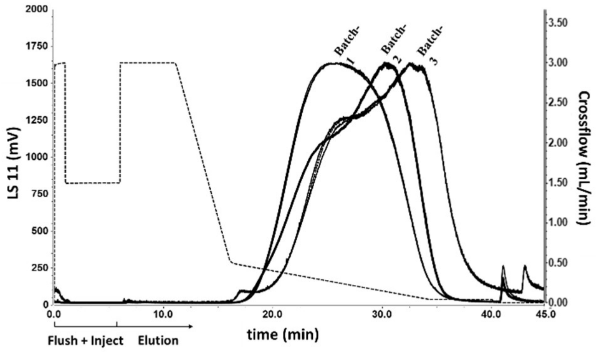

Fig. S7. Overlay of light scattering fractograms for repeats of different OMV Batches 1-3; X-axis time (min); Y-axis signal from the 90˚ light scattering detector.

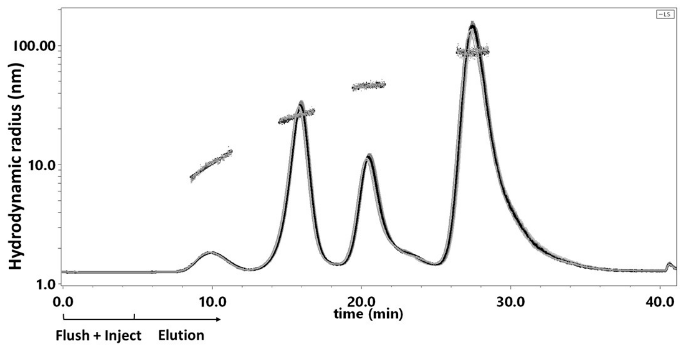

Fig. S8. Elution of particle size standards elution using method D particle standards (n=6). Thermo Fisher polystyrene particle size standard mixture, 22 nm, 51 nm, 100 nm, and 203 nm (diluted 1X, 5X, 40X, 100X respectively prior to mixing); X-axis Time (min), Y-axis Hydrodynamic radius (nm).

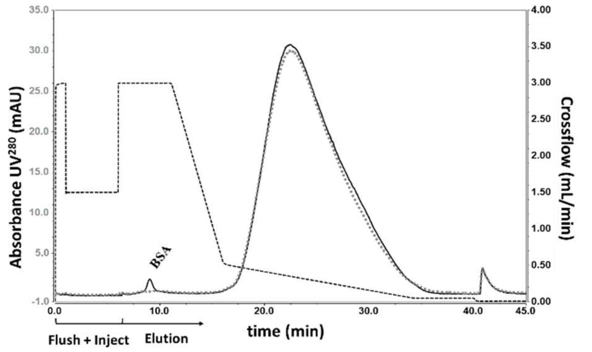

Fig. S9: Lowest BSA injection (1% or 054 µg); X-axis time (min); Y-axis left Absorbance UV^{280}, Y-axis right Crossflow (mL/min).

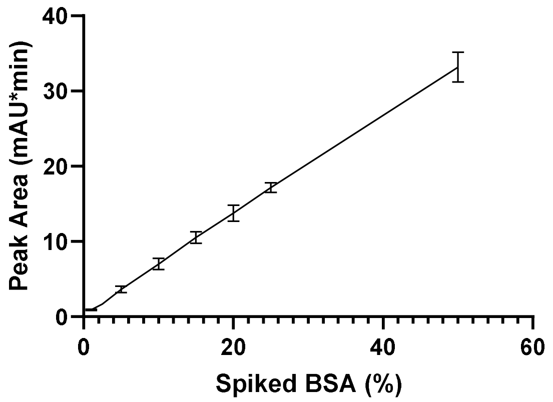

Figure S10: Triplicate overlay of correlation Spiked BSA (%) to response (Area). X-axis BSA Spiked (%); Y-axis Peak Area (mAU*min). Mean with 95% CI, R^2: 0.999.

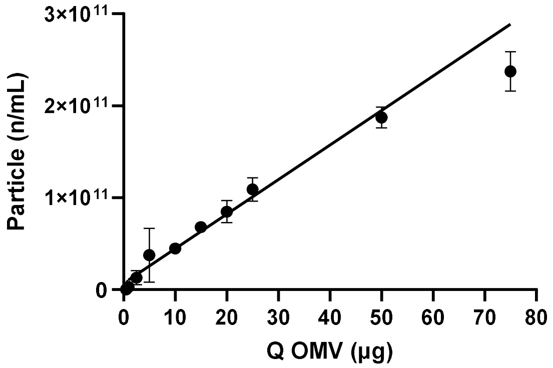

Figure S11: Triplicate overlay of correlation particle count OMV. X-axis Q_{OMV} (µg); Y-axis Particle count (n). Mean with 95% CI, R^2: 0.980. The last point in the graph was considered an outlier and as such excluded from the calculations.

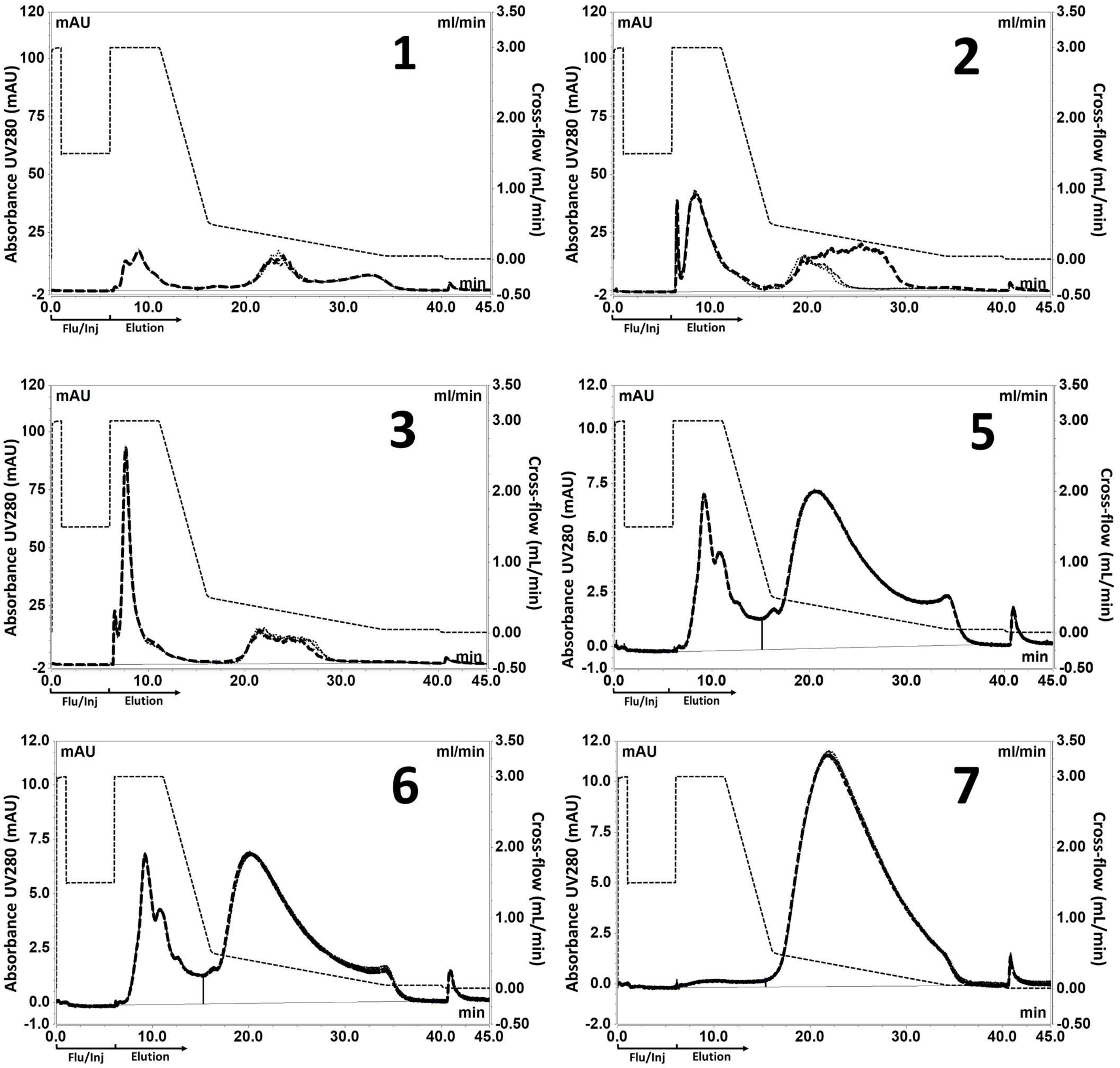

Figure S12: FFF-MALS results (n=3) for fraction 1, 2, 3, 5, 6 and 7 from the downstream purification process as described in Table 1. X-axis: time (min). Crossflow is shown as the black dotted line, of which the first 6 minutes represent the steps to flush the channel and inject the sample (Flu/Inj). Typically smaller, protein like impurities, elute in the range between 6 and 12 minutes.

III. Supporting information Methods

Method S1 FFF-MALS configurations FFF system 1 consisted of an Eclipse AF4 FFF controller (Wyatt Technology) and an Ultimate 3000 HPLC pump equipped with a UHPLC+ temperature-controlled autosampler (Thermo Fisher Scientific). FFF separation was performed at 20˚C in a thermostatic column oven (Shimadzu CTO-20AC) using the SC short FFF channel (Wyatt Technology). For online characterization we employed a DAWN HELEOS II 18-angle light scattering detector (Wyatt Technology) equipped with an embedded Wyatt QELS DLS module replacing the photodiode at MALS angle 17, an Optilab T-rEX RI detector (Wyatt Technology) for determination of concentration, and a VWD-3400 variable-wavelength UV absorption detector (Thermo Fisher Scientific) set at 280 nm. The system was controlled by Chromeleon software (Thermo Fisher Scientific) with Eclipse (Wyatt FFF module) plug-in and data were analyzed in ASTRA software 7.1.0 (Wyatt Technology).

FFF system 2, operated at Wyatt Technology, Dernbach, Germany, comprised an Eclipse NEON FFF controller (Wyatt Technology) with DCM dilution control module to increase eluting sample concentration, a 1260 Infinity II quaternary pump and a 1260 Infinity II temperature-controlled vial sampler (both from Agilent Technologies). Separation took place in a stainless-steel SC short FFF channel (Wyatt Technology). Online characterization employed a DAWN NEON 18-angle light scattering detector and an Optilab NEON RI detector (both from Wyatt Technology), and an Infinity II multi-wavelength UV detector (Agilent Technologies). The system was controlled by VISION software 3.1.0.33 and data were analyzed in ASTRA software 8.0.2.5 (both from Wyatt Technology).

The readiness or cleanliness to operate for both systems was visually confirmed on the LS-11 signal on the MALS-detector showing a minimum of 5 decimals. When 5 decimals were not obtained the system was extensively purged using a 0.5 % sodium dodecyl sulfate solution applying the night rinse option for at least 48 hours.

Method S2 BSA and particle standards Pierce bovine serum albumin (BSA), delivered in standard ampoules at 2 mg/mL, was obtained from Thermo Fisher Scientific. Nanosphere particle size standards (Thermo Fisher Scientific) included the following sizes: 20 nm (mean diameter 23 nm ± 2 nm, or radius 11.5 ± 1nm), 3020A; 50 nm (mean diameter 51 nm ± 3 nm, or radius 25.5 ± 1.5 nm), 3050A; 100 nm (mean diameter 100 nm ±4 nm, or radius 50 nm ± 4 nm), 3100A; 200 nm (mean diameter 203 nm ± 5 nm, or radius 101.5 nm ± 2.5 nm), 3200A. Both the BSA and particle standards are envisioned as the system suitability test (SST) within the method after validation.

Method S3 Nanoparticle tracking analysis A NanoSight NS500 (Malvern Instruments) equipped with an sCMOS camera module and a 488 nm laser module was used for all NTA measurements to determine particle size distributions and particle counts. With a sample loaded in the chamber, static measurements were obtained by capturing 10 measurements of 30 seconds each. In flow mode, data were acquired with a flow rate of ~2.6 µL/min, yielding a y-drift of 4.0 pixels per frame. Like the static measurements, the flow measurements consisted of 10 measurements of 60 seconds each, with an additional 5-second delay between measurements. Temperature

control was set to 25 °C. Data from both static and flow measurements were analyzed using NTA 3.2 software build 3.2.16. The capture settings of the NTA software were: Camera shutter 1300; Camera gain 512; Camera level 16; Camera hi-limit 3294; Camera lo-limit 0; Stage -57; Focus -16546. The analysis settings were: Detector threshold variable; Auto blur and Auto min track length both 'on'. The machine was calibrated by the NanoSight NTA concentration measurement upgrade. All results are reported as the mean radius (nm), which is the number-average hydrodynamic radius.

Method S4 Batch dynamic light scattering Off-line DLS measurements were performed using a Zetasizer Nano ZS (Malvern Instruments) to determine particle size. Measurements were made in disposable polystyrene semi-micro cuvettes (Greiner Bio-one, 613101) and analyzed with the Zetasizer software version 7.11. A standard operating procedure (SOP) was defined for the measurements wherein the sample was set as 'protein' (refractive index of 1.450, absorption of 0.001) and the dispersant set as 'water' (viscosity of 0.8872 cP, refractive index of 1.330). Each sample was measured three times using the measurement angle of 173° (backscatter), automatic measurement duration and "seek for optimal position" as positioning setting. Data processing was performed with the general purpose (normal resolution) analysis model. All results are reported as the harmonic 'z-average' radius R_h in units of nanometers.

Safety statement: no unexpected or unusually high safety hazards were encountered during any of the methods, processes, or assays.
